# A Colorectal Cancer 3D Bioprinting Workflow as a Platform for Disease Modeling and Chemotherapeutic Screening

**DOI:** 10.3389/fbioe.2021.755563

**Published:** 2021-11-18

**Authors:** Yordan Sbirkov, Diana Molander, Clement Milet, Ilia Bodurov, Boyko Atanasov, Radoslav Penkov, Nikolay Belev, Nico Forraz, Colin McGuckin, Victoria Sarafian

**Affiliations:** ^1^ Department of Medical Biology, Medical University of Plovdiv, Plovdiv, Bulgaria; ^2^ Research Institute at Medical University of Plovdiv, Plovdiv, Bulgaria; ^3^ CTIBiotech, Lyon, France; ^4^ UMHAT-Eurohospital, Clinical Pathology Department, Plovdiv, Bulgaria; ^5^ UMHAT-Eurohospital, Surgical Department, Plovdiv, Bulgaria; ^6^ Department of Propaedeutics of Surgical Diseases, Medical University of Plovdiv, Plovdiv, Bulgaria; ^7^ Medical Simulation Training Centre, Medical University of Plovdiv, Plovdiv, Bulgaria

**Keywords:** 3D bioprinting, colorectal cancer, drug testing, cancer modeling, chemotherapeutic screening

## Abstract

Colorectal cancer (CRC) is the third most common malignancy and has recently moved up to the second leading cause of death among carcinomas. Prognosis, especially for advanced diseases or certain molecular subtypes of CRC, remains poor, which highlights the urgent need for better therapeutic strategies. However, currently, as little as 0.1% of all drugs make it from bench to bedside because of the inherently high false-positive and false-negative rates of current preclinical and clinical drug testing data. Therefore, the success of developing novel treatment agents lies in the introduction of improved preclinical disease models which resemble *in vivo* carcinomas closer, possess higher predictive properties, and offer opportunities for individualized therapies. Aiming to address these needs, we have established an affordable, flexible, and highly reproducible 3D bioprinted CRC model. The histological assessment of Caco-2 cells in 3D bioprints revealed the formation of glandular-like structures which show greater pathomorphological resemblance to tumors than monolayer cultures do. RNA expression profiles in 3D bioprinted cells were marked by upregulation of genes involved in cell adhesion, hypoxia, EGFR/KRAS signaling, and downregulation of cell cycle programs. Testing this 3D experimental platform with three of the most commonly used chemotherapeutics in CRC (5-fluoruracil, oxaliplatin, and irinotecan) revealed overall increased resistance compared to 2D cell cultures. Last, we demonstrate that our workflow can be successfully extended to primary CRC samples. Thereby, we describe a novel accessible platform for disease modeling and drug testing, which may present an innovative opportunity for personalized therapeutic screening.

## Introduction

Neoplastic diseases are a leading cause of death worldwide, being the first or second most common reason for premature mortality in 112 of 183 countries with 10 million total fatalities in 2020. Globally, a near 50% increase in the incidence of cancer is expected by 2040. Colorectal cancer (CRC) in particular accounts for about 10% of all newly diagnosed malignancies, making it the third most frequent type of cancer in both sexes after lung and breast cancer, with nearly 2 million new patients in 2020. Despite earlier diagnosis and improved treatment strategies, the total number of CRC deaths is also rising, placing this malignancy as the second leading cause of mortality among cancer patients with more than 900 thousand fatal cases in 2020 (Sung et al., 2021). Five-year overall survival in advanced stage patients is only 7–14% (Crooke et al., 2018), which poses an urgent need for better understanding of CRC tumorigenesis and improved therapeutic strategies.

In oncology, the probability of a new candidate drug successfully passing all clinical trial phases is estimated to be merely 3.4% (Wong et al., 2019). Conventional monolayer (2-dimensional, 2D) cell culture and *in vivo* animal models have been widely used as preclinical drug testing platforms, but experience has proven that they perform poorly at predicting patient drug response and are at least in part to blame for disappointingly low translational rates of new drugs into the clinic. If the inherent failure of current *in vitro* and *in vivo* models is taken into account, then the probability of a drug passing successfully from preclinical testing to approval may be well below 0.1% (Seyhan, 2019). This leads to large economic losses, but most importantly to false-positive and false-negative new drug selection and ultimately inefficient patient treatment and unsatisfactory cure rates. Furthermore, the “right drug for the right patient” concept of personalized medicine is also failed by most existing models utilizing primary patient samples.

Current preclinical experimental systems have an inherent inability to recapitulate the complex *in vivo* tumor biology. Therefore, novel 3D culturing methods and technologies are needed in order to reproduce intercellular communication, contact with the extracellular matrix (ECM), and the intricate architecture of the tumor microenvironment (TME). The ultimate goal for innovative preclinical models is to resemble the tumor *in vivo* as closely as possible and to achieve higher predictive rates for drug response.

Spheroid and organoid cell cultures have been extensively used and have already proven the physiological and predictive superiority of 3D systems over conventional monolayer methods (Reidy et al., 2021). The next level for even more intricate and relevant drug testing platforms in oncology and personalized medicine is advancing to the biological implementation of new technologies such as microfluidics and 3D bioprinting, which are already showing promising results (Augustine et al., 2021). Despite its broader use (encompassing the 3D bioprinter use in any biology-related context), 3D bioprinting can be more accurately defined as the spatially and temporally controlled continuous deposition of layers consisting of living cells and extracellular support materials (bioink). This process of additive manufacturing is automated through a user-controlled device (a bioprinter), capable of fabricating complex three-dimensional constructs with high precision and, very importantly, with great reproducibility. Thereby, 3D bioprinting presents the opportunity to re-create the *in situ* cell niche in *in vitro*/*ex vivo* conditions by mimicking the cell’s natural microenvironment through the addition of at least some extracellular matrix components, adhesion molecules, growth and signaling factors, and stromal cells. Simulation of blood circulation, flow, and tension forces and other physiologically relevant elements can also be applied (Datta et al., 2020; Augustine et al., 2021). Last but not least, 3D bioprinters offer several key technical advantages over other methods for growing cells in 3D: unmatched uniformity between hundreds of consecutive prints, high speed (of both printing and placing cells immediately in a 3D microenvironment), and great flexibility regarding printing technologies (extrusion-based, inkjet-based, or laser-based) and bioinks (Derakhshanfar et al., 2018).

A number of studies taking advantage of 3D bioprinting have paved the way for developing the next generation of preclinical cancer models (Datta et al., 2020; Augustine et al., 2021). Extensive work in ovarian, cervical, and breast cancer shows the vast capabilities of this methodology. Xu et al. were one of the first groups to create a high-throughput 3D bioprinted cancer model using droplet-based printing of cells in suspension on Matrigel precoated plates (Xu et al., 2011). Another relatively simple example was seen with the first 3D bioprinted cervical cancer model using extrusion-based printing with HeLa cells. It demonstrated the importance of cell–cell and cell–ECM interactions for cell morphology, proliferation, expression of matrix metalloproteases, and drug response compared to 2D cultures (Zhao et al., 2014). These technologies can allow for printing of preformed spheroids for almost immediate recapitulation of the TME and drug testing (Swaminathan et al., 2019) but also can be used for more complex models. Grolman et al. developed an intricate 3D coculture system where they extruded alginate-based fibers consisting of MDA-MB-231 breast cancer cells surrounding a core of RAW 264.7 macrophages. While these fibers could be grown in standard plates, they could also be adapted to a microfluidic device. The authors demonstrated using their unique system that pharmacological targeting of macrophage migration could disrupt the spatial organization and cell interactions in their coculture fibers (Grolman et al., 2015). Zhou et al. developed another complex model using stereolithography-made scaffolds to study the interplay among breast cancer, bone, and mesenchymal stem cells (MSCs). This system showed that when interacting, cancer cells are stimulated to proliferate and secrete vascular endothelial growth factor (VEGF), while osteoblast or MSCs slow down their growth (Zhou et al., 2016). These important advances in 3D bioprinting highlight the critical role of the ECM and TME, and demonstrate the physiological and drug-testing advantages of such systems over conventional 2D cultures (Datta et al., 2020; Augustine et al., 2021). Of note, despite extensive 3D culturing work, no colorectal cancer 3D bioprinted models have been described so far (Reidy et al., 2021). Therefore, we designed a novel CRC 3D bioprinting workflow and validated it as a platform for disease modeling and individualization of therapy.

## Materials and Methods

### Cell Culture

Caco-2 colorectal cancer cells were generously donated by Associate Professor Marian Draganov at the Medical University of Plovdiv. Both Caco-2 cells and primary dissociated CRC cells (described below) were grown in DMEM/F12 supplemented with 10% FBS and 0.5% Pen/Strep (P04-41250, P40-37500, and P06-07100, PAN-Biotech, Germany) under standard cell culture conditions. The only difference in culturing the patient sample cells compared to Caco-2 cells was the higher concentrations of Pen/Strep (1–2%) and the addition of 0.5 μg/ml amphotericin B (P06-01100 PAN-Biotech, Germany). 3D bioprints were cultured in the media described above depending on whether Caco-2 or primary samples were printed.

### Collection of Patient Samples and Cell Isolation

Patient samples (Supplementary Table S1) were used after informed consent was signed. This study was approved by the Ethics Committee at the Medical University of Plovdiv, Protocol No 6/December 20, 2018. Tumor tissues were collected at the time of surgical removal of the colorectal cancers. Small pieces of the lesions (around ∼2 × 2 × 2 to 3 × 3 × 3 cm depending on the tumor size) were resected and transported in PBS to the laboratory for further tissue dissociation. In brief, each tumor was washed for 5 min in 0.5% sodium hypochlorite in PBS (similar to previously described protocols (Chao et al., 2012; Boutin et al., 2017)), rinsed in PBS twice, and cut into smaller pieces of ∼5 mm thickness. These pieces were then collected in a 15-ml tube and incubated for >30 min with rotation in 2.5 ml collagenase IV and 0.5 ml dispase (catalog # 07909 and 07923, STEMCELL Technologies, Canada). The dissociated cells were then centrifuged, enzymes were removed, and the cells and tissue pieces were washed at least twice in PBS prior to cell culturing.

### 3D Bioprinting

The computer-aided design (CAD) of the model was made with Tinkercad (Autodesk, Canada). The diameter of the model was set to 5 mm, and the height was set to 0.82 mm (2 layers of 0.41 mm which is the gauge of the nozzle). For printing in the 96-well plates, the same model was used, but the diameter was reduced to 2 mm. Slicing of the model was performed by the inbuilt software of the BioX extrusion–based bioprinter (CELLINK, Sweden) prior to printing. We used a CELLINK RGD bioink (catalog # IK1020100301, CELLINK, Sweden), composed of alginate with covalently bound RGD and nanofibrillar cellulose, with viscosity of 3–20,000 Pa/s and shear rate 0.002–500 1/sec (taken from the manufacturer’s specification sheet). In brief, the bioprinting process would start by detaching Caco-2 or primary CRC cells using Accutase® (catalog # 423201, BioLegend, United States). Cells were then counted, centrifuged to remove all supernatant, and mixed with the CELLINK RGD bioink (containing alginate with covalently bound RGD and nanofibrillar cellulose at 30 million cells/ml for Caco-2 cells and 5–20 million cells/ml for CRC samples (depending on the patient sample)) in sterile 3 ml cartridges (catalog # CSC010311101, CELLINK, Sweden). The cell–bioink mixture was then extruded through a 410-μM (0.41 mm) high-precision nozzle (catalog # NZ3220005001, CELLINK, Sweden) at 8–10 kPa pressure, 10 ms speed (with 200 ms preflow delay) in standard 24-well cell culture plates. This protocol would yield up to ∼50 prints from 1 ml of bioink and cells. Bioprints were then crosslinked for 1 min with CaCl_2_ (catalog # 1010006001, CELLINK, Sweden), washed twice with PBS, and left with 2 ml DMEM/F12 medium (as described previously) in the incubator. Media were replaced every 2–3 days for at least 2 weeks while all assays were performed.

### Calcein AM/PI Cell Viability Assays

To assess cell viability after 3D bioprinting, we used calcein AM (catalog # 56,496, Sigma-Aldrich, United States) and propidium iodide (catalog #P4170, Sigma-Aldrich, United States). In brief, 3D bioprints were incubated with calcein AM (at 5 ng/ml final concentration) for >10 min at 37°C, and then PI (at 2 μg/ml final concentration) was added. The prints were then manually cut using scalpels and tweezers into thin slices on a microscopic slide, and the conversion of calcein AM and incorporation of PI were assessed using a fluorescent microscope (Nikon Eclipse Ni, Japan) at 490/525 nm and 580/600 nm, respectively.

### Drug Titrations and MTT Tests

The day prior to drug titrations, Caco-2 cells were split with Accutase® (catalog # 423201, BioLegend, United States) and seeded at 5,000 cells/well in 96-well plates. Cells in triplicates were then treated for 3 days with nine different concentrations of each of the chemotherapeutics: irinotecan, 5-fluoruracil (5-FU), and oxaliplatin (catalog #I1406-50MG, F6627-1G, and O9512-5MG, Sigma-Aldrich, United States) to determine IC_50_ values. On day 3, MTT (methylthiazolyldiphenyl-tetrazolium bromide-catalog #M5655-500MG, Sigma-Aldrich, United States) was added to each well at a final concentration of 0.5 mg/ml and incubated for 2–4 h at 37°C. Finally, DMSO was added to a final concentration of 20%, and plates were read at 592 nm. Experiments were repeated at least twice, and data were analyzed and plotted using GraphPad Prism 9.

Drug testing in 3D bioprints was carried out from day 12 to day 14 (for 3 days) after bioprinting in order to allow for cells to form and grow larger aggregates and to match the time point of the RNA-seq analysis in the 12-well plates. The MTT test was performed analogously to the test in 2D Caco-2 cultures with one modification: since the MTT formazan crystals remained in the prints, the cell medium was removed completely and DMSO was added to each well. Cells were then placed on a shaker for 15–30 min to dissolve and release the crystals. The DMSO–extracted dye from each well was then transferred to a 96-well plate and measured at 592 nm. These experiments were carried out in biological replicates and technical duplicates.

### Histology and Immunohistochemistry

Paraffin-embedded 3D bioprints or primary CRC samples fixed in 10% neutral buffered formaldehyde were sectioned (5 μM) and mounted on microscopic slides for further analysis. Sections were deparaffinized and hydrated, and then the standard hematoxylin and eosin (H&E) staining was carried out for pathomorphological assessment. For immunohistochemistry of cytokeratin 20 (CK20), after dewaxing and hydration, an antigen unmasking step was carried out by incubating samples in a water bath in a preheated 0.1 M citrate buffer (pH 6.0) for 20 min at 96°C. Samples were then washed 3 times each with PBS-glycine and ddH_2_O. Endogenous peroxidase was then blocked (3% H_2_O_2_ in methanol for 30 min) followed by a step of blocking nonspecific binding with 1% BSA in PBS for 30 min. A primary rabbit anti-CK20 antibody (catalog # NBP1-85, Novus Biologicals, United States) was diluted 1:100 in 1% BSA in PBS and incubated overnight at 4°C on the slides. On the following morning, after 3 washes with the blocking solution, the sections were stained with Vectastain Universal Quick Kit, ImmPACT NovaRED, as a chromogen and counterstained with hematoxylin QS (catalog # VE-PK-7800, VE-SK-4805, and VE-H-3404, Vector laboratories, United States) following the protocol recommended by the manufacturer with one additional blocking of endogenous peroxidase as suggested in the standard protocol.

### RNA Isolation and RNA-Sequencing

For RNA extraction, >1 million Caco-2 cells grown as monolayers were accutased, collected, and pelleted in 1.5 ml tubes in duplicates. Six 3D bioprints were transferred into two 1.5 ml tubes. Both cell pellets and bioprints were washed twice with PBS, and the RNA was isolated using Qiagen RNeasy Kit (Qiagen, United States) according to the manufacturer’s protocol. No modification of the protocol was required for RNA isolation of bioprints besides longer and more vigorous vortexing of the samples with the RLT lysis buffer until no pieces of the bioink could be observed visually. Sample concentration and quality were measured by using Nanodrop (Thermo, United States), and the frozen RNA was sent to Novogene, United Kingdom, for further QC and processing for RNA sequencing. Samples were reverse-transcribed after which NEBNext® UltraTM RNA Library Prep Kit for Illumina® (NEB, United States) was used for library preparation. Size selection and PCR purification were carried out with AMPure XP beads (Beckman Coulter, Beverly, United States), following the instructions of the manufacturer. Finally, sequencing was performed on an Illumina instrument, and all samples yielded >20 M pair-end reads each.

### Data Analysis and Statistics

HISAT2 software, DESeq2, and EdgeR package in R were used by Novogene for quality control, mapping, quantification, and differential gene expression analysis. Thresholds for adjusted *p*-value of ≤0.05 and fold change (FC) of ≥1.5 and ≤-1.5 were applied in the generated lists with differentially expressed genes for further analyses—gene ontology (GO) analysis with BinGO (Maere et al., 2005) in Cytoscape software (Shannon, 2003) and gene set enrichment analysis (GSEA) with GSEA software by the Broad Institute (Subramanian et al., 2005) (using standard settings of 1,000 permutations, gene_set as the permutation type, and the H: hallmark gene set from the molecular signature database (with FDR ≤0.05)). Correlation analysis between differentially expressed genes in the TCGA “COAD” (colon adenocarcinoma, *n* = 271) sample group (vs control tissue, *n* = 41) and 3D bioprinted Caco-2 cells (vs cells grown in 2D) was carried out in GEPIA2 (Tang et al., 2019) following the instructions set by the developers. Data analysis and figure preparation of the results from MTT assays were carried out with GraphPad Prism (v.8). Datasets from at least 2 separate experiments in technical triplicates were analyzed together by normalizing the values, transforming the drug concentrations to logarithmic scale (log10), and using nonlinear regression to fit the titration curves and calculate IC_50_ values. For bar chart figures, the same data from the MTT assays normalized to control (Figure 3A) and another set of data obtained from 3D bioprints as described previously (Figure 3B) were used. Student’s t-tests were run for every treatment group comparison. The image counting of live/dead cells was carried out manually using ImageJ (NIH, United States). All cells in an area of approximately 1 mm^2^ were counted from at least 2 representative images. Mean with SD was plotted using GraphPad Prism (v.8).

## Results

### Cell Viability and Morphology of Caco-2 Cells in 3D Bioprints

**FIGURE 1:**
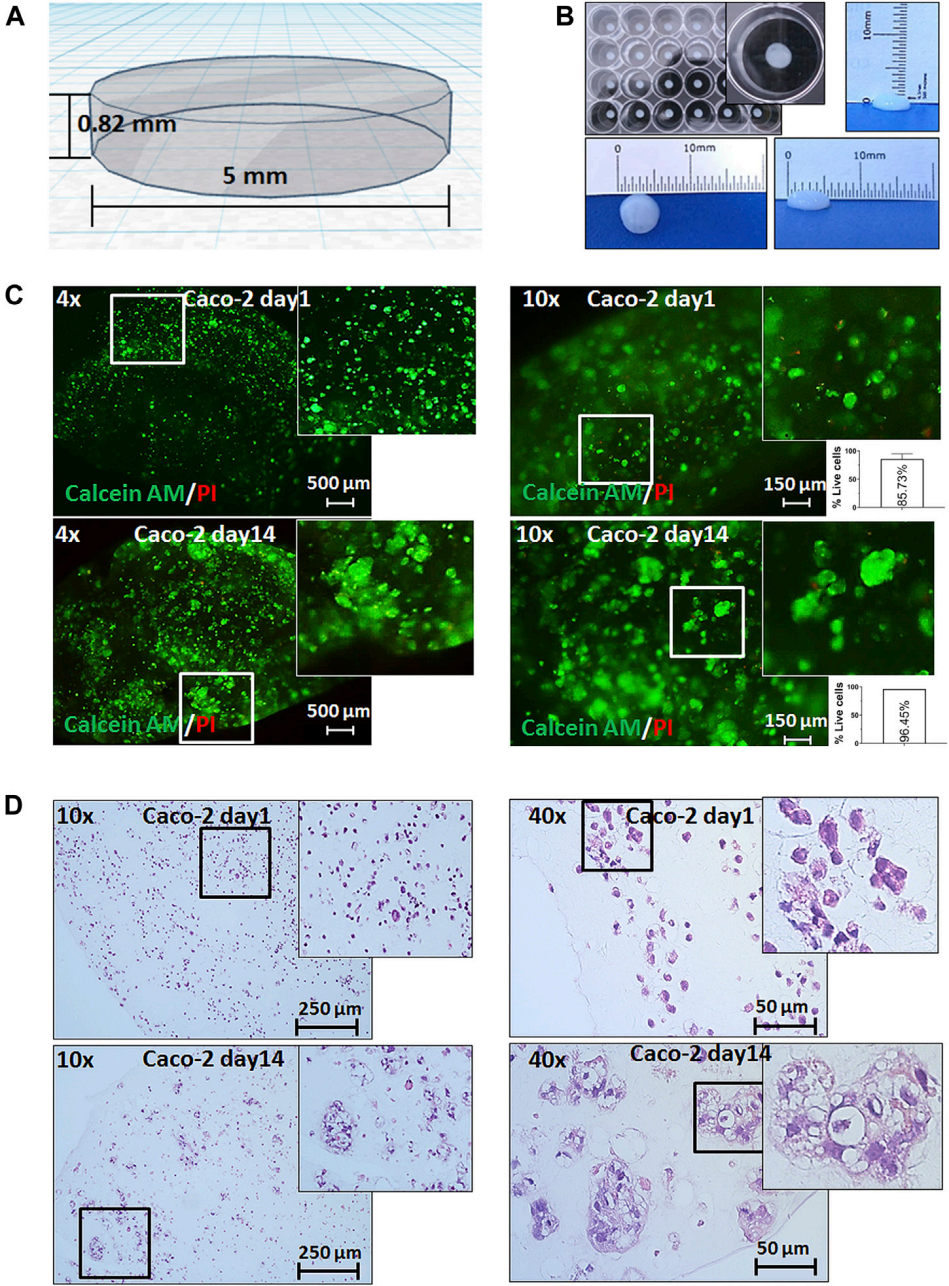
3D bioprinting of Caco-2 cells. **(A)** Computer-aided design (CAD) of the developed 3D bioprinting model. **(B)** Representative macroscopic image of 3D bioprinted cells in a 24-well plate showing the reproducibility and uniformity of prints (with the growth medium) and of the size of a 3D bioprint. **(C)** Live/dead staining with calcein AM (green fluorescence) for live cells and propidium iodide (PI-red fluorescence) for dead cells on day 1 and day 14 after printing under magnifications as shown, and relative quantification of percentage live cells (per 1 mm^2^). Error bars represent SD of the mean from at least two images. **(D)** Hematoxylin and eosin (H&E) staining of 3D bioprinted Caco-2 cells on day 1 and day 14 with magnifications as annotated showing the formation of tumor-like morphological structures of cells at day 14.

We designed a simple two-layer cylindrical 3D model of 0.82 mm height and 5 mm diameter. The small dimensions of this 3D bioprinting model aimed to fulfill two critical tasks: (i) first, to ensure that nutrients and drugs can easily reach all cells within the print, and (ii) second, to offer reproducibility in large numbers of prints allowing testing of multiple drugs (Figures 1A,B and Supplementary Figure S1A). Caco-2 colorectal cancer cells were detached from their flasks, put in suspension, and were mixed with a commercially available bioink containing the common ECM peptide motif RGD (R-arginine, G-glycine, and d-aspartic acid). The resulting mixture of cells and bioink was extruded in multiple uniform prints of two layers, crosslinked with calcium chloride (CaCl_2_), and grown in the standard medium.

We first assessed the viability and distribution of cells through the prints. It was found that cells displayed an even spread within the prints, survived for more than four weeks embedded in our constructs, and showed insignificant cell death throughout this follow-up period (Figure 1C and Supplementary Figure S1B). Importantly, monitoring cells for their viability allowed assessment of cell growth and/or migration over time. We observed the formation of larger structures starting from day 3 (data not shown) which became more visible at weeks 1 and 2 (Figures 1C,D). In parallel, we performed histological analysis which confirmed the cell viability and distribution within the bioprints. The H&E staining and histopathological assessment revealed that the morphology of these larger cell clusters closely resembled small glandular-like structures similar to those observed *in situ* in CRC.

### Transcriptomic Analysis of 3D Bioprints vs 2D Monolayer Cells

**FIGURE 2:**
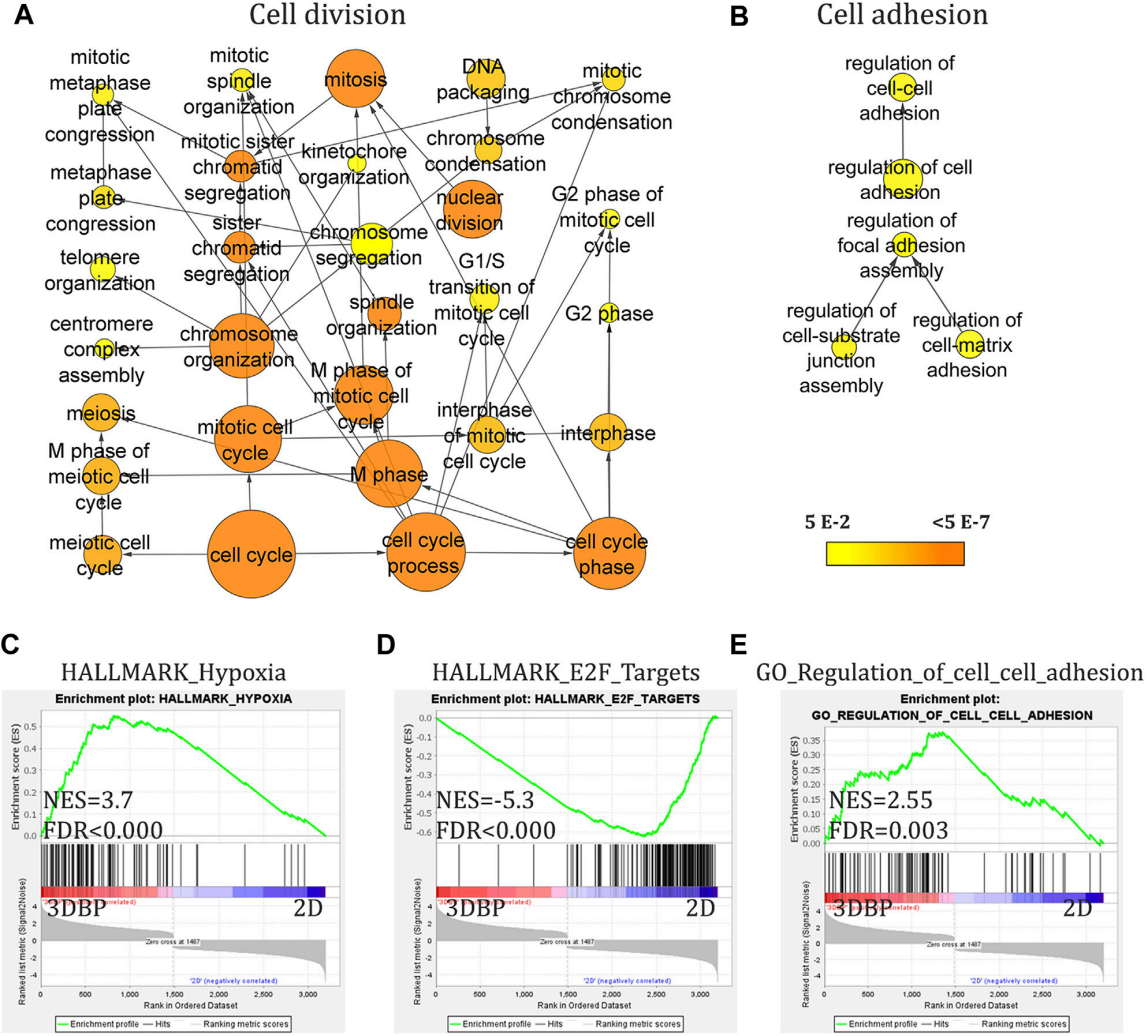
RNA-seq analysis of 3D bioprinted and 2D monolayer Caco-2 cells. **(A)** and **(B)** Gene ontology (GO) analysis (with BinGO in Cytoscape, cutoff 1.5-fold change and adj. *p*-value<0.05) of all differentially expressed genes showing nodes involved in cell cycle regulation and of upregulated genes in 3D bioprinted (3DBP) cells showing nodes for cell adhesion. **(C–E)** Gene set enrichment analysis (GSEA) of differentially expressed genes (cutoff 1.5-fold change and adj. *p*-value<0.05) demonstrating enrichment for genes involved in hypoxia and cell–cell adhesion. (**(C)** and **(E)**) Negative enrichment (downregulation) of genes involved in cell cycle progression/control.

After the initial characterization of our model for viability and morphology, we asked how the 3D environment of the bioprints affects the cells compared to standard 2D cultures. We used 14-day-old prints of Caco-2 cells for RNA-seq analysis, as at this point, cells would have formed and grown large aggregates and compared gene expression to the same cell line, but cultured in the monolayer. We found that growth in 3D bioprints can strongly and significantly alter the transcription of over 3200 genes (cutoff at 1.5 fold change and padj ≤0.05). Initial gene ontology (GO) analysis of all differentially expressed genes showed a significant representation of nodes containing genes involved in nucleic acid metabolism (corrected *p*-value = 1.62 × 10^−46^), cell cycle control (corrected *p*-value = 1.39 × 10^−41^), apoptosis (corrected *p*-value 3.47 × 10^−3^), and others (Figure 2A, Supplementary Figure S2 and Supplementary Table S2). Further analysis suggested the origin of these GO nodes. We found that a significant number of genes upregulated in 3D bioprinted cells are involved in response to stress (corrected *p*-value 5.15 × 10^−8^), to oxygen levels (corrected *p*-value 6.86 × 10^−6^) and hypoxia (corrected *p*-value 4.74 × 10^−5^), and regulation of cell adhesion, but also apoptosis and cell cycle arrest. The genes downregulated in 3D bioprinted cells appeared to be involved mainly in DNA repair and DNA replication (Supplementary Table S2 and Supplementary Figure S2).

Furthermore, GSEA analysis continued to reveal the changes in 3D bioprints vs 2D cultures. We found several key patterns of expression, which could be expected and also validate the physiological relevance of the model, namely, cells in 3D bioprints augment transcriptional programs for cell–cell adhesion and hypoxia. Interestingly, GSEA also showed activation of some of the most prominent pathways in CRC–EGFR, K-RAS, and TNFα (*via* NFkB). Of note, 3D bioprinting appeared to negatively affect cell cycle and replication (E2F targets and progression through G2M checkpoint), and also possibly viability (apoptosis) compared to 2D conditions (Figure 2C-E and Supplementary Figure S3A-E).

Last, we investigated if the 3D bioprinted model is physiologically relevant by comparing our RNA-seq data to publically available datasets from TCGA. By using the online platform GEPIA2 (Tang et al., 2019) and its correlation analysis, we found that the upregulated genes in 3D bioprints correlate significantly (*R* = 0.51, *p* < 0.000) to the upregulated genes in these patient samples. The downregulated genes (or upregulated in 2D) showed more mild correlation (*R* = 0.35, *p* = 3.6 × 10^−9^) (Supplementary Figure S3F,G).

### 3D Bioprints as a Platform for Colorectal Cancer Drug Testing

**FIGURE 3:**
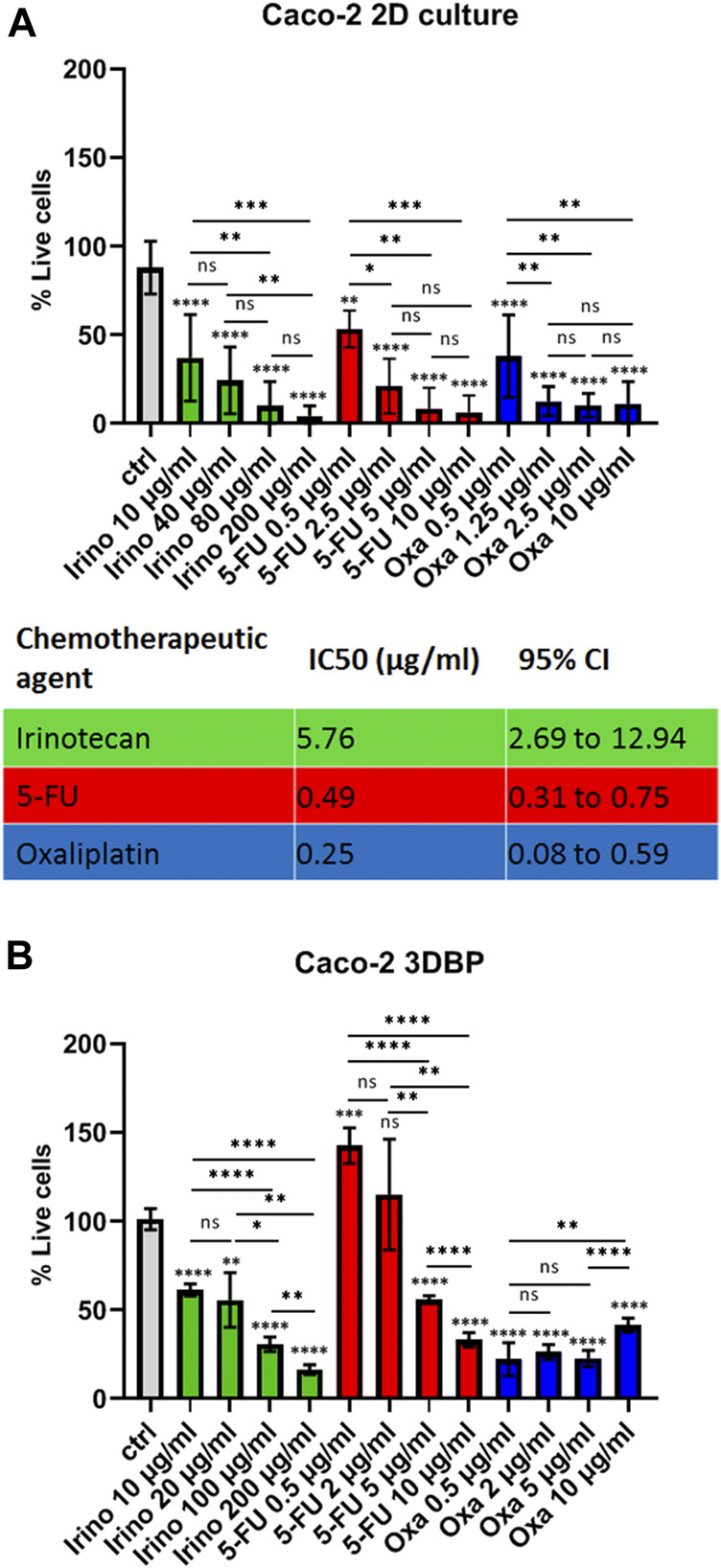
Drug response in 2D cells vs 3D bioprinted cells. **(A)** Response of Caco-2 cells grown in monolayers (2D) to different concentrations of irinotecan, 5-fluoruracil (5-FU), and oxaliplatin as annotated and a summary table of the respective IC_50_ values. Cells were seeded and treated on the next day with the inhibitors for 72 h. Cell viability was measured with the MTT test, and IC_50_ values were calculated from at least 2 biological replicates in technical triplicates. IC_50_ values and 95% confidence interval values are both in ug/ml. **(B)** Response of 3D bioprinted cells (biological duplicates in technical duplicates) to 4 concentrations of the 3 inhibitors as annotated. Caco-2 cells were bioprinted (as described in Methods) and grown for 2 weeks prior to drug treatment. Error bars represent mean with SD. *p ≤ 0.05, **p ≤ 0.005, ***p < 0.0005, and ****p < 0.0001, ns: not significant, Student’s *t*-test. Asterisk symbols immediately above error bars indicate significance compared to control samples; lines with symbols show significance between the relevant treatment groups.

The next question we asked was if 3D bioprinting and the changes in gene expression would translate to alterations in cell response to standard chemotherapy. Therefore, we tested three of the most commonly used chemotherapeutics in CRC—5-fluoruracil (5-FU), oxaliplatin, and irinotecan as single agents on both monolayers of Caco-2 cells and on 3D bioprints. Only oxaliplatin remained effective at similar concentrations in 2D and 3D cells, while the other two chemotherapeutics failed to reproduce similar IC_50_ values to the ones previously determined on standard monolayer cultures, namely, 3D bioprinted cells showed ∼2.4-fold resistance to irinotecan (estimated IC_50_ = 14.05 μg/ml, 95% CI = 10.26–19.06 for 3D bioprinted cells, compared to IC_50_ = 5.76 μg/ml, 95% CI = 2.69–12.94 for cells in monolayer) and ∼6-fold resistance to 5-FU (estimated IC_50_ = 3 μg/ml, 95% CI = 2.4–3.74 for 3D bioprinted cells, compared to IC_50_ = 0.49 μg/ml, 95% CI = 0.31–0.75 for cells in 2D) (Figure 3, Supplementary Figure S4 and data not shown). Thereby, we demonstrated that the 3D bioprinted model of Caco-2 cells is markedly different from standard 2D cultures in both gene expression patterns and drug response.

### 3D Bioprinting Method Validation in Colorectal Cancer Patient Samples

**FIGURE 4:**
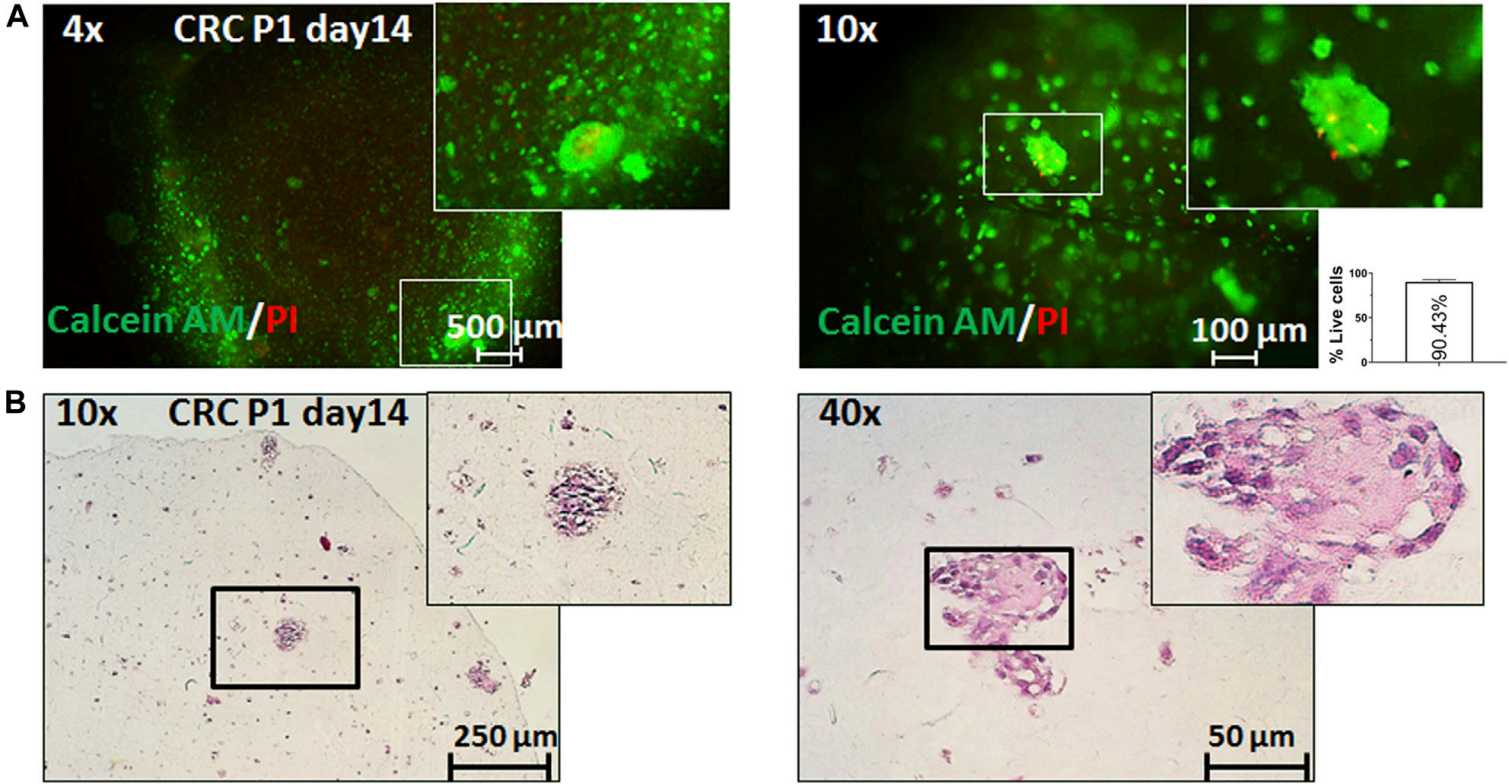
3D bioprinting of primary CRC samples. Representative pictures of 3D bioprinted primary CRC cells taken on day 14 after printing. **(A)** Live/dead staining with calcein AM (live cells: green fluorescence) and propidium iodide (dead cells: red fluorescence) under ×4 and ×10 magnification and relative quantification of percentage live cells (per 1 mm^2^). Error bars represent SD of the mean from at least two images. **(B)** Representative pictures of the hematoxylin and eosin (H&E) staining of sections from 3D bioprinted primary cells under ×4 and ×10 magnification.

Last, we sought to validate the proposed model on patient samples. Cells from resected CRC were isolated, cultured, and expanded in sufficient numbers for bioprinting (see Methods). Even though growth and expansion of primary CRC cells over extended periods of time are difficult, we managed to produce several million cells and to print them (Figure 4). Similar to what we observed with Caco-2 cells, these primary samples showed good viability over extended periods of time (>2 weeks), and, importantly, we could see clusters of cells with malignant histopathological appearance and expression of epithelial cell surface markers (Supplementary Figure S5). Of note, the size of the primary printed tumors on day 14 was comparable to that of Caco-2 cells (>50 μm), implying similar growth rates and thereby good reproducibility and translatability of our printing workflow to patient samples.

## Discussion

3D bioprinting has so far been used by multiple groups to develop both homotypic (containing one type of cells like in the model) and more complex heterotypic models, implementing a variety of 3D bioprinting methods (mostly extrusion-based, but also droplet-based, laser, and stereolithographic). The focus on malignancies, however, has been almost exclusively on breast, ovarian, and brain cancers (Satpathy et al., 2018; Datta et al., 2020; Augustine et al., 2021). Besides a vast spectrum of 3D cell culturing methods (including various spheroid techniques (Reidy et al., 2021)), the closest work done in the context of CRC prior to this study included the following: the use of alginate (micro)beads enclosing HCT-116 cells to assess viability, tumor markers (Rios de la Rosa et al., 2018), and drug response (Shakibaei et al., 2015); printing of collagen-based scaffolds on which HCT-116 cells could be grown (together with stromal cells) (Chen et al., 2020); bioprinting of bovine colon cells (in GelMa bioink) (Töpfer et al., 2019); and the biofabrication of a human small intestine model (again for drug penetration and toxicity studies) (Madden et al., 2018). Furthermore, the literature on bioprinting of primary cancer patient samples is scarce, with one recent proof-of-concept work including two hepatocellular carcinoma samples and one sarcoma sample (Maloney et al., 2020).

Therefore, we have taken important first steps toward 3D bioprinting of CRC by proposing a widely accessible workflow with several key advantages. First, this method is highly reproducible from both biological and technical point of view due to the even distribution of cells in the ink and the small variations in the size of the bioprints, which we attribute to both the excellent printability of this particular bioink and to the precision of the bioprinter (Figure 1). Second, this workflow can be easily adjusted not only in terms of cell types and numbers but also in volumes of the prints and printing formats (Supplementary Figure S1C, D). Third, and most importantly, we show that this model is markedly different from standard 2D cultures in its morphology, transcriptional programs, and response to drugs which has long been the criticism of 2D cultures. Determining whether these differences are partially due to the biochemical and biophysical properties of the hydrogel itself, or to the altered cell–cell interactions within the bioprints, or to both factors was, unfortunately, beyond the scope of this study. This original experimental platform could provide more physiologically relevant settings to study CRC development and to address current drug testing concerns.

False-positive or false-negative drug response data in preclinical models have been a major setback in drug transitioning to the clinic. Therefore, a number of 3D bioprinting studies have focused on implementing their cancer models for proof-of-concept assessment of the effect of certain widely used inhibitors. The paramount roles of the ECM and 3D cell growth in drug response and resistance were perhaps first demonstrated more than 10 years ago. Loesnner et al. showed that two ovarian cancer cell lines (OV-MZ-6 and SKOV-3) embedded in an RGD-containing hydrogel matrix loose sensitivity to paclitaxel by 2–3-fold (Loessner et al., 2010). Similarly, bioprinted HeLa cells in fibrinogen/gelatin/alginate bioink scaffolds have shown several-fold resistance to paclitaxel (Zhao et al., 2014). Bioprinted glioma cell lines (U87 and glioma stem cells SU3) have also acquired resistance (∼2-fold) to temozolomide compared to 2D cultures (Dai et al., 2016). In a more complex 3D bioprinted breast cancer model containing three types of stromal cells (fibroblasts, endothelial cells, and pre-adipocytes) and MCF-7 cells, Langer et al. demonstrated that 3D bioprinted cells were 20-fold and ∼5000-fold more resistant to doxorubicin and paclitaxel, respectively, than 2D cocultures of these 4 cell lines, again suggesting the significant impact of 3D-organization and/or ECM interactions (Langer et al., 2019).

In the context of novel preclinical drug testing platforms and personalized medicines, our 3D bioprinted model can be easily readjusted to allow screening in 96-well plates without compromising the reproducibility of the bioprints (Supplementary Figure S1C, D). The time required to print in this format (at 15 ms speed) is only about 10 min, which further boasts the capabilities of this technology. However, even if the volume of the bioprints can be reduced significantly, the concentrations of cells per ml and the total number of cells required for an experiment remain the biggest challenges for tuning this platform for the needs of personalized medicine. While the technical flexibility and ease of use are critical prerequisites for wider implementation of this methodology and potential replacement of other *in vitro* drug testing systems, the more important aspect of this study is related to the biological changes we observed. The key findings in our 3D bioprinted Caco-2 cell model are the drug resistance to irinotecan and 5-fluoruracil and the gene expression alterations in 3D bioprinted cells compared to monolayer cultures. Of note, we did not observe increased resistance to oxaliplatin, and the reason for this may lie in its mode of action. Oxaliplatin exerts its ubiquitous inhibitory effect on cancer cells by forming stable adducts between the two strands of DNA, thus blocking both replication and transcription (Kelland, 2007). Being a third-generation platinum-based drug, this chemotherapeutic has proven to be much more effective than carboplatin and cisplatin (Martinez-Balibrea et al., 2015). Intrinsic resistance to this chemotherapeutic can be considered rare, and much few possible resistance mechanisms to oxaliplatin compared to 5-FU and irinotecan have been described (Jeught et al., 2018). Even though further in-depth investigation of the exact mechanisms of resistance may be required, it is tempting to speculate that certain transcriptional patterns may well be the underlying cause of resistance to irinotecan and 5-FU.

From all altered gene expression programs we found in 3D bioprinted cells, perhaps the most obvious explanation for drug resistance would be the observed negative enrichment for cell cycle progression (E2F and G2/M targets–Figure 2 and Supplementary Table S2). The cell cycle delay is widely accepted as a common drug resistance mechanism as most chemotherapeutics interfere with DNA replication targeting the rapidly dividing bulk of cancer cells (and sparing quiescent cells). In CRC, there are several comprehensive reviews on the subject which illustrate the significance of cell cycle perturbations in multiple drug resistance (Shah and Schwartz, 2001; Briffa et al., 2017; Blondy et al., 2020) as well publications directly linking 5-FU resistance to cell cycle dysregulation (Kim et al., 2020). Hypoxia is another more general and nonspecific mechanism for drug resistance (e.g., through drug efflux, metabolic reprogramming, and driving of genetic instability) that could explain our results (Velázquez et al., 2016; Briffa et al., 2017). TNFα (*via* NF-κB) signature is enriched in 3D bioprinted cells as well (Supplementary Figure S3A). This signaling pathway is commonly activated in CRC (Yu, 2004) and has been strongly implicated in drug resistance to irinotecan and 5-FU (Scartozzi et al., 2007; Cascinu et al., 2008; Shakibaei et al., 2015).

EGFR–RAS signaling is activated in 3D bioprinted cells as well (Figure 2, Supplementary Figure S3 and Supplementary Table S2), and this pathway is perhaps the most commonly dysregulated one in CRC, with ∼40% of patients presenting with KRAS mutations (and up to 10% having NRAS or BRAF mutations) (Inamura, 2018). Caco-2 cells do not harbor KRAS (or NRAS) mutations, so possible explanations of the enrichment for this pathway could be the upregulation of several components in the signaling cascade. We found a 2.1-fold increase of the expression of ERB-b3 (HER3) (adj. *p* = 2.05 × 10^−6^), a 7.1-fold upregulation of FOSB (adj. *p* = 8.19 × 10^−8^), a 3.9-fold increase of JUN (adj. *p* = 1.43 × 10^−20^), and a modest upregulation of BRAF (∼1.55-fold change, adj. *p* = 0.0085). Importantly, RAS is a downstream mediator of other non-EGFR family cell surface molecules as well. Integrin binding can also activate the RAS-ERK axis. We found that several genes encoding such adhesion molecules are upregulated in 3D bioprinted cells. Of note, ITGB6 (3.3 fold change, adj. *p* = 3.98 × 10^−8^) has been shown to mediate resistance to 5-FU in CRC (Liu et al., 2013). Analogously, TIMP2 (a tissue inhibitor of metaloproteinases 2) is also upregulated in our model (1.5-fold change, adj. *p* = 5 × 10^−4^). It has been shown that high expression of this molecule in CRC patients is related to 5-FU resistance likely through the activation of ERK (Zhang et al., 2020). Last, this pathway and ultimately ERK activation have been linked not only to cell proliferation and poorer overall survival rates but also to resistance to other chemotherapeutics, especially in hematopoietic malignancies (Abrams et al., 2010).

Data mining in the literature followed by a targeted search in our gene lists found that several genes directly implicated in drug resistance in CRC cells and validated in patient survival data (Zheng et al., 2015) are differentially expressed in our 3D bioprinted cells. For 5-FU resistance out of 13 upregulated and 14 downregulated genes listed by Zheng et al., we found that 4 (ABCC3, FOX O 3, LGALS3, and SULF2) were upregulated, while 5 (SLC19A1, FANCA, RRM1, MTAP, and NF2) were downregulated in our dataset (30 and 35%, respectively). ABCB1 was also upregulated (1.9-fold, adj. *p* = 1.9 × 10^−5^) in 3D bioprinted cells, and this gene codes for a pump widely implicated in 5-FU resistance (Wang et al., 2015; Blondy et al., 2020). For irinotecan (or its active form SN-38), we found 6 upregulated genes (SRC, CSF1R, LOX, ERBB3, SAT1, and LDLR) out of 22 listed genes, and only 1 downregulated gene (MSH3) out of 24 listed (27 and 4%, respectively). Last, for oxaliplatin, we detected only 2 upregulated genes out of 14 (CD24 and RICTOR) and 5 downregulated genes (DHFR, EFIF4E, LYN, TYMS, and MTAP) out of 30 (14 and 17%, respectively). Therefore, besides the aforementioned altered pathways, some of these specific transcriptional changes could provide another explanation for drug resistance in Caco-2 3D bioprinted cells.

Our novel 3D bioprinting workflow of CRC presents an intriguing experimental platform for answering fundamental questions about carcinogenesis. The model would be even more valuable if it can contribute toward precision medicine, and successfully printing primary CRC cells derived from three tumors all in different stages (Supplementary Table S1) proves the broad applicability of the workflow. The main limitation of this study is the proof-of-concept validation in primary patient samples due to several technical issues. In our hands, expanding these primary CRC cells to large enough numbers for bioprinting (i.e., >10 million cells) was difficult mainly because of bacterial contaminations (arising from the gut flora). The lack of standardized medium conditions that would provide stable and prolonged proliferation of the primary cells is another constraint which did not allow for drug testing in patient-derived 3D bioprints. Therefore, our success rate of expanding primary samples was merely about 15%. Furthermore, we found that the aggregation of cells into morphologically more relevant structures in the prints is related to the number of printed cells, and this is another consideration when working with patient samples.

Even if we validated and proved that the printed cells are indeed of epithelial origin and grow in a very characteristic way in the culture (Supplementary Figure S5), genetic testing (e.g., through short tandem repeats, STR, and profiling) would be the ideal method to verify the relevance of the model. Further phenotyping for the presence of stromal cells from these patient-derived samples may also be useful as the role of the TME in CRC progression and drug response is well-established (Klemm and Joyce, 2015; Reidy et al., 2021). In this context, multiple groups have highlighted the importance of complex cocultures for chemotherapy resistance (e.g., in breast cancer (Wang et al., 2018a; Langer et al., 2019) and glioma (Wang et al., 2018b)). The addition of layers with other cell types is the next step toward the 3D reconstruction of CRC *in vitro*, but this was beyond the scope of our work. Nevertheless, mimicking the TME has been given a successful start with 3D modeling of ovarian (Loessner et al., 2010), breast (Langer et al., 2019), and other types of cancer (Datta et al., 2020), and it is a matter of time for 3D bioprinting of CRC to follow these encouraging examples.

In summary, we present a simple, highly reproducible, and flexible 3D bioprinted model of CRC which resembles certain aspects of the cells closer than conventional 2D cultures. We validated our workflow as a template for testing the response to chemotherapeutics. Therefore, this novel *in vitro* model has the potential to become a useful experimental platform for future fundamental and preclinical studies directed to personalized medicines.

## Data Availability

The raw files and the processed data from RNA-seq, which were used for the analyses in this work can be found in the Gene Expression Omnibus, GEO under accession number GSE181722 https://www.ncbi.nlm.nih.gov/geo/.
