# Supplementary material for: A Colorectal Cancer 3D Bioprinting Workflow as a Platform for Disease Modeling and Chemotherapeutic Screening
Source: Front Bioeng Biotechnol. 2021 Nov 18;9:755563. doi: 10.3389/fbioe.2021.755563 (PMC8638705; doi:10.3389/fbioe.2021.755563)
Supplement: Supplementary file 1 [file DataSheet1.docx]

Supplementary Material

# Supplementary Figures and Tables

## Supplementary Figures

**Supplementary Figure 1**


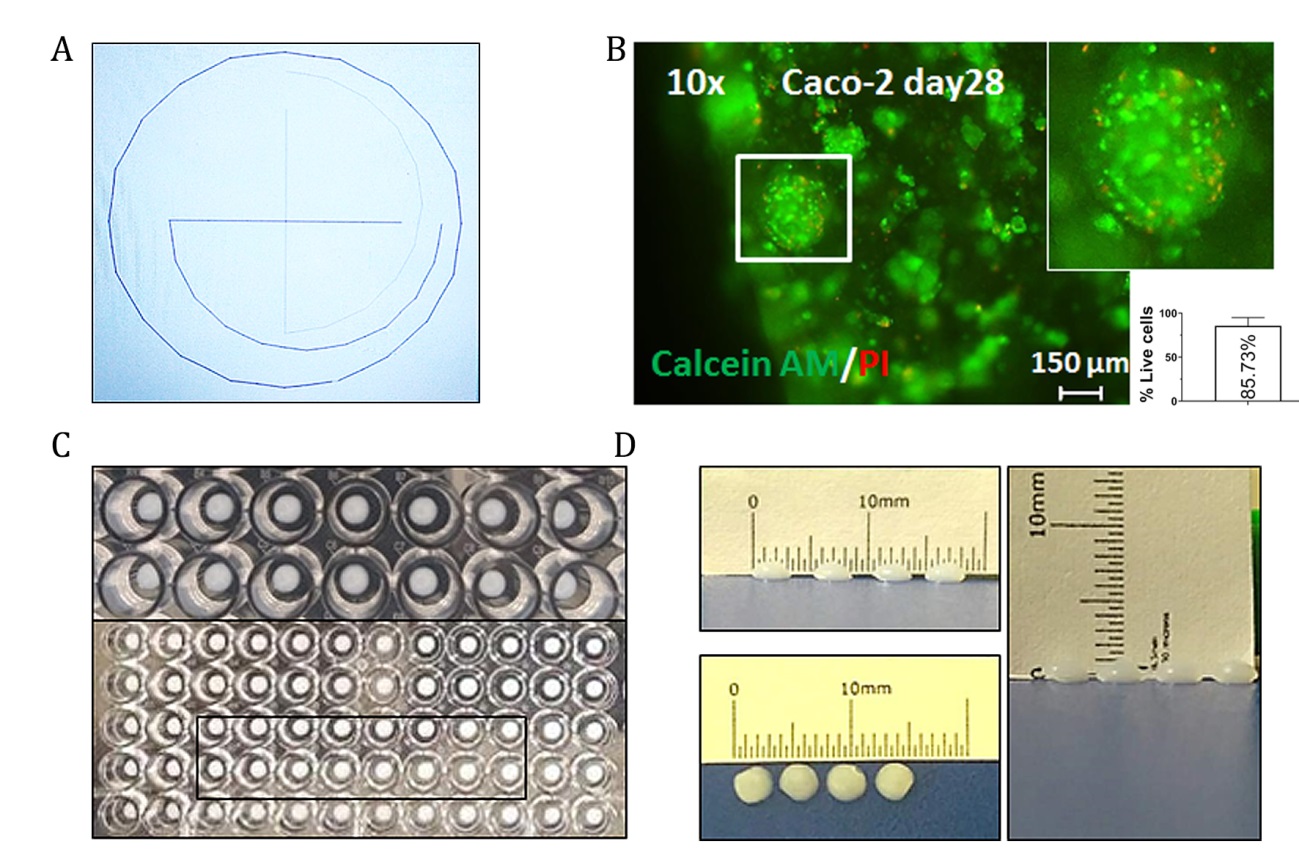


A) Slicing of the 3D model (Figure 1A) carried out automatically by the BioX software. The two layers of printing are shown in grey and blue (infill density was set to 25%, with grid printing pattern). B) A representative image of Caco-2 cells at day 28 after printing as annotated and relative quantification of percentage live cells. Error bars represent SD of the mean of % live cells counted in ~1mm2 from at least 2 representative images. C) and D) Representative images of the reproducibility of printing in a 96-well format with readjusted 3D model to diameter of 2mm and infill density of 15%.

**Supplementary Figure 2**

**
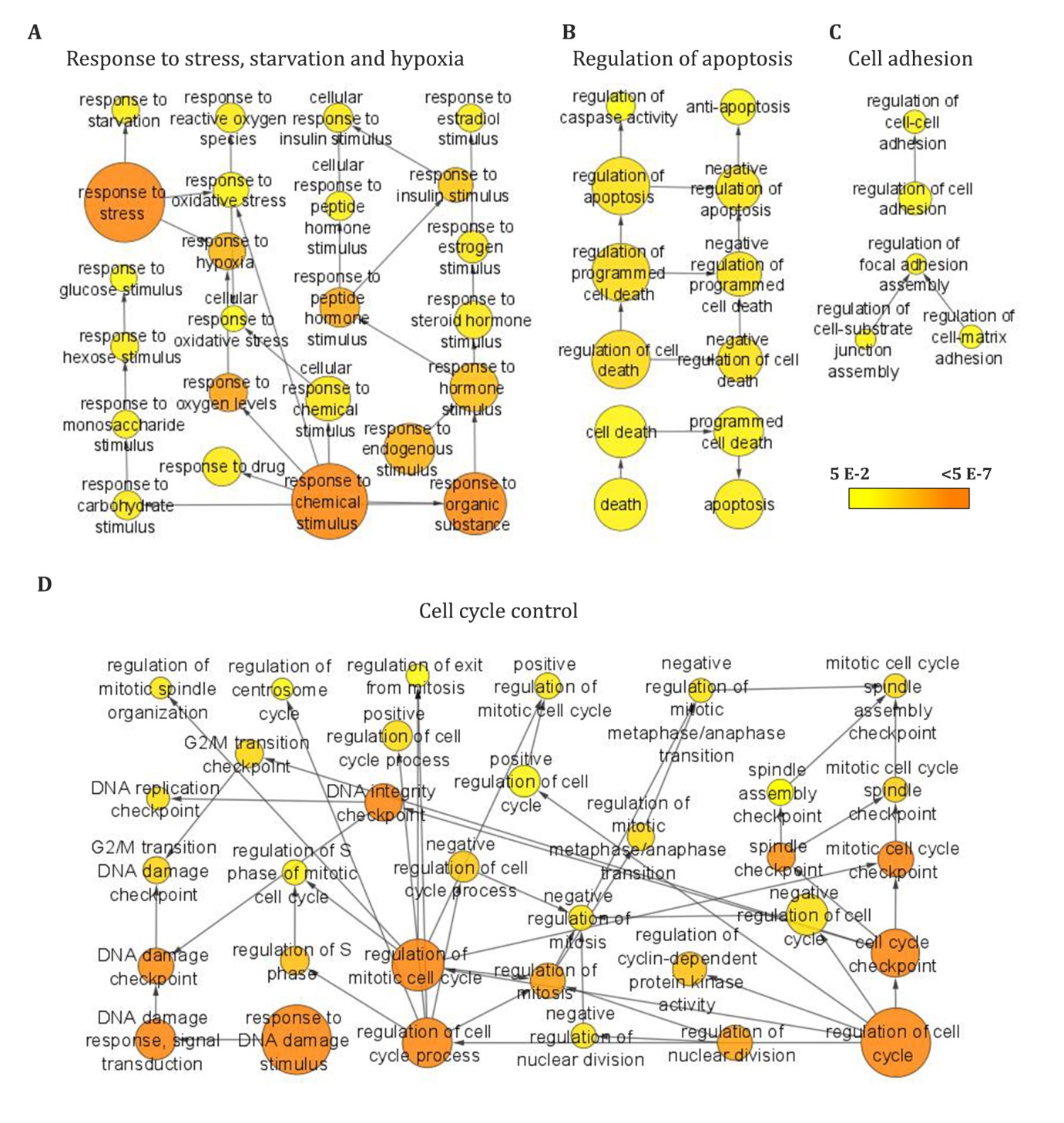
**

Gene ontology (GO) analysis for biological processes (BinGO in Cytoscape) displaying enrichment of the upregulated genes in 3D bioprinted cells in nodes involved in **A)** response to stress (e.g. oxygen deprivation, hypoxia), **B)** apoptosis and **C)** cell adhesion. **D)** Genes which were downregulated in 3D bioprinted cells showed enrichment in nodes for cell cycle control.

**Supplementary Figure 3**


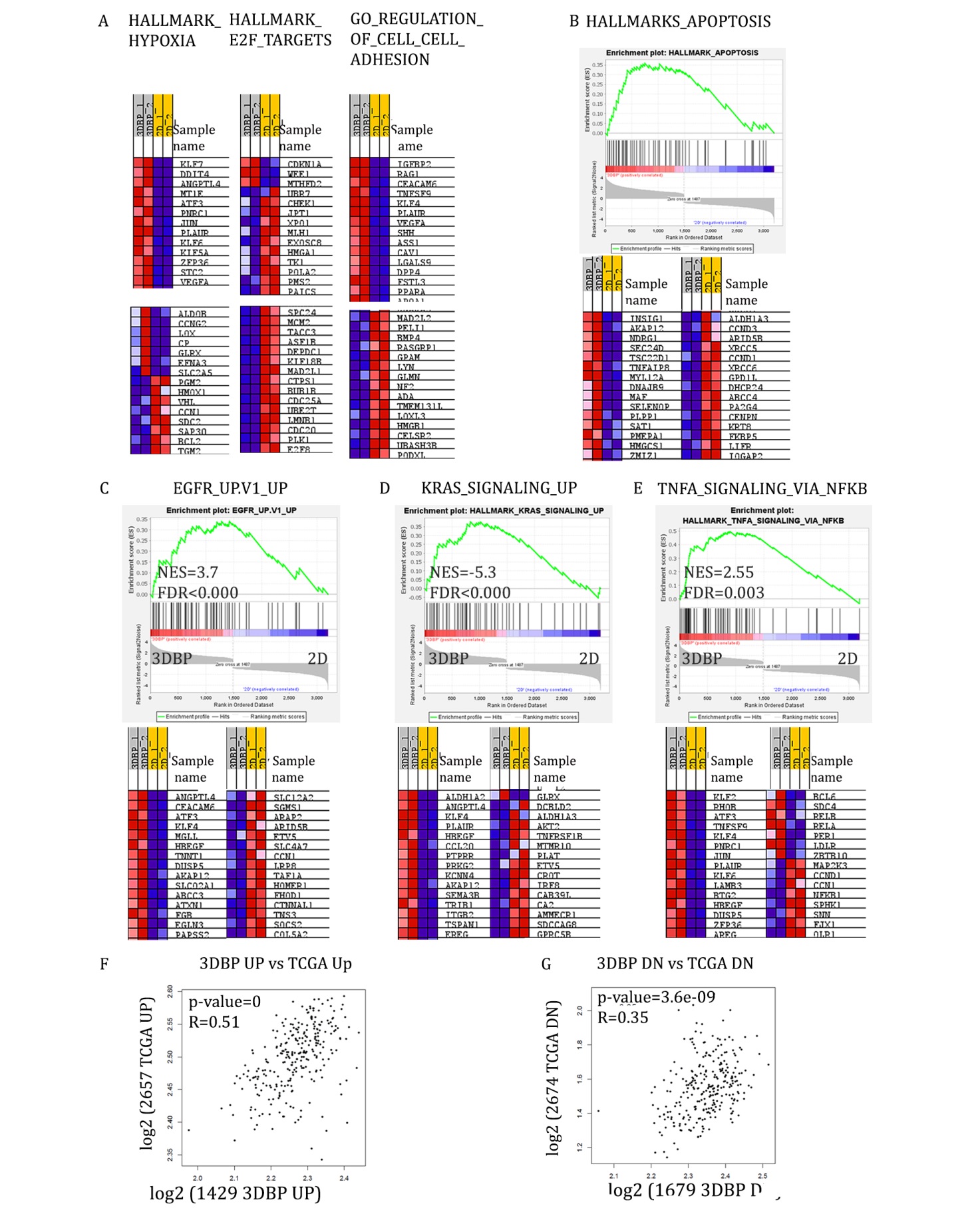


Gene expression changes in Caco-2 cells in 3D bioprints compared to standard 2D cultures were analysed via RNA-sequencing. Subsequent Gene Set Enrichment Analysis (GSEA) was carried out. **A)** Heatmaps of the top and bottom 15 genes that showed enrichment for hypoxia, cell cycle (E2F targets) and cell adhesion. **B-E)** Enrichment plots and top 15 up- and down-regulated genes involved in apoptosis, EGFR, K-RAS and NF-kB signalling as annotated. Correlation analysis of all upregulated **(F)** and downregulated **(G)** genes in the 3D bioprints compared to respectively up- and down-regulated genes in the TCGA colon adenocarcinoma (COAD) cohort. TPM,   Transcripts per million.

**Supplementary Figure 4**


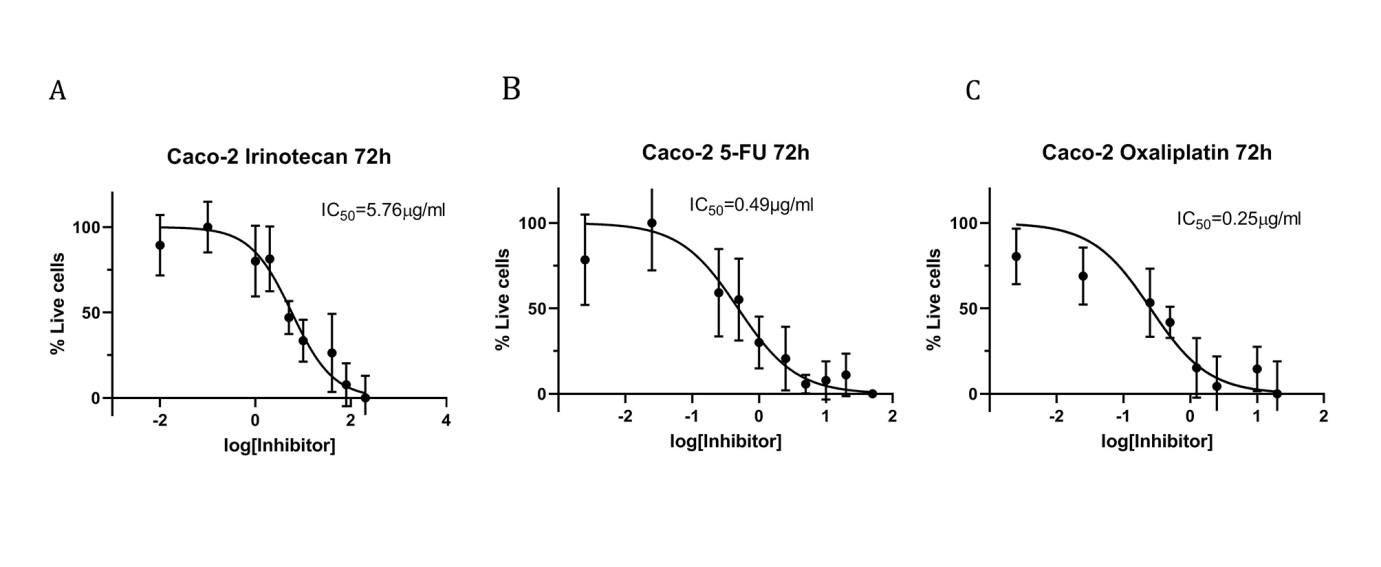


Caco-2 cells were grown in standard monolayer (2D) culture and treated with **A)** irinotecan, **B)** 5-fluoruracil (5-FU), or **C)** oxaliplatin for 72h and IC_50_ values were calculated (using GraphpadPrism). Error bars represent standard deviation from two experiments in technical triplicates.

**Supplementary Figure 5**

**
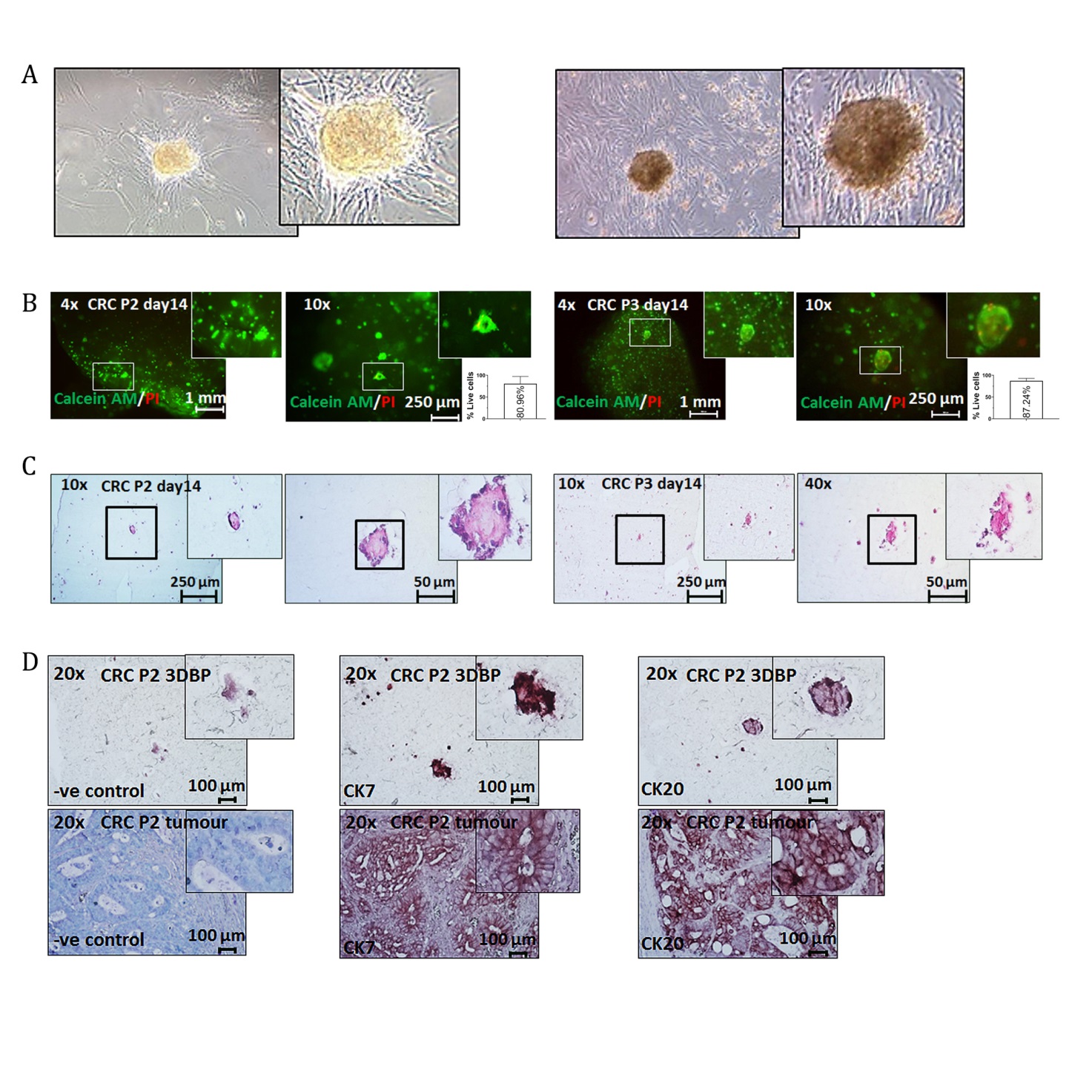
**

**(A)** Representative images of primary CRC cells grown in culture (10x magnification). **B)** Live/Dead staining with Calcein (green-live cells) and Propidium Iodide (PI-red-dead cells) and relative quantification of percentage live cells. Error bars represent SD of the mean of % live cells counted in ~1mm2 from at least 2 representative images. **C)** Hematoxylin and Eosin (H&E) staining of 2 primary patient samples (CRC P2 and CRC P3) on day 14 after printing as annotated. **D)** Immunohistochemical validation of the epithelial origin of the printed cells (top panel) comparing levels of Cytokeratin 7 and 20 (CK7 and CK20) in bioprints (top panel) and primary tumour section (bottom panel) as annotated.

## Supplementary Tables

**Supplementary Table 1.**

**Patient information**

| Patient sample # | Diagnosis | Age | Gender | Stage (AJCC) |
| --- | --- | --- | --- | --- |
| CRC P1 | Colorectal cancer | 54 | Female | III A |
| CRC P2 | Colorectal cancer | 80 | Female | IIA |
| CRC P3 | Colorectal cancer | 68 | Male | IIIB |

**Supplementary Table 2**

**Selected GO and GSEA hits**

| **GO Description** | | **corrected p-value** |
| --- | --- | --- |
| **GO of all differentially expressed genes** | |  |
| nucleobase, nucleoside, nucleotide and nucleic acid metabolic process | | 1,62E-46 |
| nucleic acid metabolic process | | 1,22E-44 |
| cell cycle | | 1,39E-41 |
| cell cycle process | | 2,93E-36 |
| cell cycle phase | | 3,81E-36 |
| M phase | | 3,29E-34 |
| cellular metabolic process | | 5,70E-31 |
| apoptosis | | 3,47E-03 |
| response to drug | | 1,41E-02 |
|  | |  |
| **GO of Upregulated genes in 3DBP cells** | |  |
| response to stress | | 5,15E-08 |
| response to oxygen levels | | 6,85E-06 |
| response to hypoxia | | 4,74E-05 |
| negative regulation of cell proliferation | | 6,48E-04 |
| regulation of cell death | | 1,42E-03 |
| regulation of apoptosis | | 2,52E-03 |
| regulation of focal adhesion assembly | | 1,74E-02 |
| regulation of cell-substrate junction assembly | | 1,74E-02 |
| regulation of cell-cell adhesion | | 1,77E-02 |
| anti-apoptosis | | 1,98E-02 |
| cell-substrate junction assembly | | 2,14E-02 |
| regulation of cell adhesion | | 2,66E-02 |
| blood vessel development | | 2,18E-03 |
| angiogenesis | | 5,70E-03 |
| response to drug | | 6,24E-03 |
|  | |  |
| **GO of Downregulated genes in 3DBP cells** | |  |
| double-strand break repair | | 1,86E-06 |
| double-strand break repair via homologous recombination | | 4,86E-04 |
| recombinational repair | | 4,86E-04 |
| base-excision repair | | 1,38E-03 |
| regulation of DNA replication | | 3,60E-06 |
| regulation of mitosis | | 6,67E-06 |
| G1/S transition of mitotic cell cycle | | 1,39E-05 |
| G2/M transition DNA damage checkpoint | | 7,84E-04 |
| regulation of cyclin-dependent protein kinase activity | | 1,28E-04 |
| cell proliferation | | 2,02E-03 |
|  |  |  |
| **GSEA analysis of all differentially expressed genes** |  |  |
| NAME | NES | FWER p-value |
| HALLMARK_HYPOXIA | 3.71 | 0.0 |
| HALLMARK_TNFA_SIGNALING_VIA_NFKB | 3.06 | 0.0 |
| HALLMARK_MYOGENESIS | 2.45 | 0.0 |
| HALLMARK_CHOLESTEROL_HOMEOSTASIS | 2.25 | 0.005 |
| HALLMARK_KRAS_SIGNALING_UP | 2.23 | 0.009 |
| HALLMARK_APOPTOSIS | 2.14 | 0.019 |
| HALLMARK_INFLAMMATORY_RESPONSE | 2.14 | 0.02 |
| HALLMARK_COAGULATION | 2.12 | 0.021 |
| HALLMARK_HEME_METABOLISM | 2.04 | 0.038 |
| HALLMARK_BILE_ACID_METABOLISM | 2.03 | 0.039 |
|  |  |  |
| HALLMARK_E2F_TARGETS | -5.33 | 0.0 |
| HALLMARK_G2M_CHECKPOINT | -4.73 | 0.0 |
| HALLMARK_MYC_TARGETS_V1 | -3.54 | 0.0 |
| HALLMARK_MYC_TARGETS_V2 | -2.79 | 0.0 |
| HALLMARK_MITOTIC_SPINDLE | -2.77 | 0.0 |
| HALLMARK_SPERMATOGENESIS | -2.25 | 0.005 |
| HALLMARK_DNA_REPAIR | -2.21 | 0.008 |

NES-normalised enrichment score; FWER-family-wise error rate
